# Deficiency of Dietary Fiber Modulates Gut Microbiota Composition, Neutrophil Recruitment and Worsens Experimental Colitis

**DOI:** 10.3389/fimmu.2021.619366

**Published:** 2021-02-23

**Authors:** Sj Shen, Kathryn Prame Kumar, Shu Wen Wen, Raymond Shim, Brooke J. Wanrooy, Dragana Stanley, Robert J. Moore, Thi Thu Hao Van, Remy Robert, Michael J. Hickey, Connie H. Y. Wong

**Affiliations:** ^1^ Centre for Inflammatory Diseases, Department of Medicine, School of Clinical Sciences at Monash Health, Monash Medical Centre, Monash University, Clayton, VIC, Australia; ^2^ School of Health Medical and Applied Sciences, Central Queensland University, Rockhamptom, QLD, Australia; ^3^ Infection and Immunity Program, Monash Biomedicine Discovery Institute and Department of Microbiology, Monash University, Clayton, VIC, Australia; ^4^ School of Science, Royal Melbourne Institute of Technology (RMIT) University, Melbourne, VIC, Australia; ^5^ Monash Biomedicine Discovery Institute and Department of Physiology, Monash University, Clayton, VIC, Australia

**Keywords:** neutrophils, colitis, fiber, inflammation, diet

## Abstract

Ulcerative colitis is an inflammatory disease of the colon that is associated with colonic neutrophil accumulation. Recent evidence indicates that diet alters the composition of the gut microbiota and influences host–pathogen interactions. Specifically, bacterial fermentation of dietary fiber produces metabolites called short-chain fatty acids (SCFAs), which have been shown to protect against various inflammatory diseases. However, the effect of fiber deficiency on the key initial steps of inflammation, such as leukocyte–endothelial cell interactions, is unknown. Moreover, the impact of fiber deficiency on neutrophil recruitment under basal conditions and during inflammation *in vivo* is unknown. Herein, we hypothesized that a fiber-deficient diet promotes an inflammatory state in the colon at baseline and predisposes the host to more severe colitis pathology. Mice fed a no-fiber diet for 14 days showed significant changes in the gut microbiota and exhibited increased neutrophil-endothelial interactions in the colonic microvasculature. Although mice fed a no-fiber diet alone did not have observable colitis-associated symptoms, these animals were highly susceptible to low dose (0.5%) dextran sodium sulphate (DSS)-induced model of colitis. Supplementation of the most abundant SCFA, acetate, prevented no-fiber diet-mediated enrichment of colonic neutrophils and colitis pathology. Therefore, dietary fiber, possibly through the actions of acetate, plays an important role in regulating neutrophil recruitment and host protection against inflammatory colonic damage in an experimental model of colitis.

## Introduction

Ulcerative colitis (UC) is a chronic and relapsing inflammatory condition with an unknown cause. Patients with UC present with debilitating clinical symptoms, including fatigue, weight loss, increased stool frequency, and bloody diarrhea, which can severely hinder daily routines (1, 2). Tissue damage observed in patients with UC is restricted to the mucosal layer of the colon and is largely the result of an abnormal immune response (3). UC has low penetrance (around 10–20% concordance in monozygotic twins) (4), suggesting that environmental factors play a more prominent role than genetic alterations in its initiation and progression. Indeed, one of the risk factors for UC and other inflammatory diseases is the consumption of a “Western” diet. Specifically, the decreased intake of fiber has been proposed as the main culprit to the increasing incidence of inflammatory diseases (5, 6). There are emerging studies that demonstrated the mechanisms underlying how immune responses and the development of inflammatory diseases can be altered by a fiber-deficient diet. We have previously shown that a no-fiber diet exacerbated clinical and pathological signs of experimental colitis, whereas another study found no worsening of experimental asthma (7, 8). These studies indicate that the deleterious effects of a diet lacking fiber are likely restricted to the local colonic environment as opposed to systemic effects on the immune response. Despite this, the exact underlying mechanisms of how a diet lacking fiber disrupts homeostasis and promotes inflammation prior to disease onset remain largely unknown.

One possible explanation for the beneficial effects of dietary fiber on general health is through its effects on the gut microbiota. Specific bacterial strains within our gut microbiota, such as *Bifidobacterium* and *Lactobacillus*, are needed to ferment fiber to produce short-chain fatty acids (SCFAs) (9). Amongst the SCFAs, acetate is the most abundant in the colonic lumen and in the circulation (8, 10, 11). As such, mice fed a diet lacking fiber had reduced abundance of the aforementioned bacteria and reduced production of SCFAs (7, 8). These studies clearly demonstrate an intimate and bilateral relationship between dietary fiber intake and gut microbiota composition, such that a possible mechanism for the anti-inflammatory effects of fiber is through the actions of SCFA-producing microbiota. Indeed, there have been supportive evidence to demonstrate that SCFAs induce the number and function of regulatory immune cells, as well as promote intestinal integrity, the full extent of which has been reviewed elsewhere (11). Accordingly, supplementation of individual or a mixture of SCFAs in addition to a control diet has shown promise as a prevention strategy for colitis and other inflammatory diseases (7, 8, 12). Nonetheless, there is a gap in knowledge on how the nature of gut microbiota changes over time following adoption of a new diet, and whether these changes impact on the immune system and host response to inflammation.

There is currently no single perfect experimental model that completely recapitulate human UC, because patients with UC present a heterogeneous spectrum of pathological features that reflect the participation of a diverse range of innate and adaptive immune effectors. Despite this, a key pathological feature of both clinical and experimental colitis is the significant migration of neutrophils into the colonic mucosa (13, 14), suggesting that dysregulated neutrophil recruitment contributes to the manifestation of disease. Prior research has shown that mice given a diet deficient in fiber had a dysbiotic microbiota and exacerbated dextran sodium sulphate (DSS)-induced intestinal inflammation (7). However, it remains unclear whether the beneficial effects of dietary fiber or SCFAs on colitis are mediated through factors that control neutrophil recruitment, such as chemokines and their receptors, or vascular adhesion molecules. In this study, we fed mice a no-fiber diet to examine the role of dietary fiber in the recruitment and function of neutrophils under basal conditions and following DSS-induced colitis. In addition, we investigated whether the changes induced by a fiber-deficient diet can be prevented by exogenous administration of the SCFA acetate. Furthermore, because SCFAs have been shown to act on neutrophils (15), we explored potential mechanisms underlying the effect of acetate on neutrophil recruitment in experimental colitis.

## Materials and Methods

### Mice

Six- to seven-week-old male C57BL/6J mice (wildtype; WT), and *LysM^eGFP^* neutrophil reporter mice [eGFP driven by the LysM promoter in mice bred on a C57BL/6J background (16)] were bred and kept under specific pathogen-free (SPF) conditions at the Monash Medical Centre (MMC) Animal Facility. Following transportation, mice were acclimatized for a minimum period of 7 days before use. All mice were housed in groups of no more than five animals in each cage after weaning, in a 12-hour light-dark cycle and a temperature-controlled environment. Autoclaved water and food pellets (Irradiated Rat and Mouse, Specialty Feeds, Australia) were provided *ad libitum*, and cages were changed weekly, unless otherwise specified. All experimental procedures were performed in accordance with protocols approved by the MMC B Animal Ethics Committee (MMCB/2015/15 and MMCB/2019/24).

### Diet and Acetate *Feeding*

Adult mice were provided with either an experimental control diet (CON; AIN93G diet with 4.7% dietary fiber content; irradiated; SF09-091, Specialty Feeds) or a no-fiber diet (NF; modified AIN93G diet with 0% dietary fiber content; irradiated; SF09-028 Specialty Feeds) for two weeks *ad libitum*. Details of the diets can be found in **Supplemental Table 1**. Mice were fed experimental diets for an additional one week for experiments utilizing the DSS model of intestinal inflammation.

To supplement acetate in the daily intake, 200 mM of sodium acetate (Sigma) in was added to the drinking water and mice had access to it *ad libitum*, as per previous studies (7, 8). Acetate was supplemented into the drinking water in combination with the diet regime and refreshed twice every week. Control mice that received no supplementation of acetate were provided with normal drinking water.

### *Fecal DNA* Extraction *and 16S rRNA* Gene Amplicon Sequencing *and* Bioinformatics

Fecal samples were collected directly from mice into individually labelled tubes in sterile conditions. The samples were immediately snap frozen in liquid nitrogen and stored at −80°C. Extraction of fecal DNA was performed using the Isolate II Genomic DNA Kit (Bioline, USA) in accordance with manufacture’s protocol. PCR (30 cycles), using 50 ng of fecal-derived DNA as template, was performed using Q5 DNA polymerase (New England BioLabs) with a primer set selected to amplify the V3–V4 region of the gene encoding the 16S rRNA (forward: ACTCCTACGGGAGGCAGCAG; and reverse: GGACTACHVGGGTWTCTAAT). The 16S rRNA gene primers also included additional barcode and spacer sequences and sequences to adapt the amplicons to Illumina sequencing, following the method of Fadrosh et al. (17). Equal quantities of each amplicon were pooled, and sequencing was performed on an Illumina MiSeq (2 × 300 bp), following the manufacturer’s protocol. Data analysis was performed using QIIME 1.9.1 software (18), and sequences were joined using the fastq-join method. The maximum allowed percentage difference within the overlapping region was zero. Sequences were de-multiplexed using the QIIME split library protocol, keeping only sequences with a Phred quality score >20. The data set was inspected for chimeric sequences using Pintail (19). Operational taxonomic units (OTUs) were picked using the UCLUST algorithm (20), and the taxonomy was assigned against the GreeneGene database (21). OTUs were picked at 97% similarity cut-off, and OTUs with less than 0.01% abundance and those assigned to Cyanobacteria were filtered out. Further data analysis was done using Calypso (22). Other than UniFrac and alpha diversity measures that used rarefied data, all statistical analysis was carried out using the OTU table that was log 2 transformed and Cumulative Sum Scaling (CSS) normalized (23).

### Mouse Model of Intestinal *Inflammation*

Adult male mice were provided with 0.5–2% (w/v) dextran sodium sulphate (DSS; molecular weight 36–50 kDa, MP Biomedicals) in autoclaved drinking water for a maximum of 7 days and weighed and monitored daily. The disease activity index (DAI) was performed as previously described (24). Clinical scoring included general symptoms of activity (0: normal, 1: isolated, 2: huddled/inactive, 3: moribund), coat (0: normal, 1: rough, 2: unkempt, 3: severe hair loss/bleeding), and dehydration (0: none, 1: skin less elastic, 2: skin tenting, 3: skin tenting and eyes sunken), and colitis-specific symptoms of percentage weight loss from the previous day (0: none, 1:0–3, 2:3–6, 3:6–10, and 4:>10%), stool consistency (0: normal, 1: soft, 2: loose, 3: liquid, 4: diarrhea), and stool blood content (0: none, 1: visible blood in stool).

### *Colon* Morphology and Histology Scoring

At the experimental endpoint, mice were anesthetized with isoflurane, and blood collected using cardiac puncture. A midline incision was made in the abdominal cavity to isolate and excise the colon, and colon length was measured. The distal colon was collected and fixed in 10% formalin for 24 h, paraffin-embedded, cut into 4 µm sections and stained using hematoxylin and eosin (H&E). Images of the colon sections were taken at 4x magnification using the Leica DM LB widefield microscope and MC120 HD camera (Leica), and scored in a blinded manner. Parameters for histology scoring were as previously described (24). Following collection of the distal colon for histology, the rest of the colon was used for flow cytometric analysis or sectioned into two parts for myeloperoxidase (MPO) assay (middle section) and RNA isolation (proximal section).

### Colon Intravital *Microscopy*

To examine the neutrophil-endothelial cell interactions in the colon during DSS-induced colitis, intravital multiphoton microscopy of the intact colon was performed on *LysM^eGFP^* mice. Mice were anesthetized by intraperitoneal injection of a mixture of 10 mg/kg xylazine hydrochloride and 150 mg/kg ketamine hydrochloride, with tail vein cannulation as described (25). Body temperature was maintained using a heat pad. Mice were placed in a supine position, and the colon exteriorized through a midline incision. All exposed tissues were kept moist with saline-soaked gauze to prevent dehydration. The colon was carefully placed between a custom 3D-printed platform and coverslip setup, as previously described (24). Vacuum grease was applied in between the 3D platform and the coverslip, allowing the colon to sit within a sealed chamber, enabling continued superfusion with a solution of 10^−2^ M atropine in saline.

At least three post-capillary venules of approximately 35–60 µm in diameter were chosen per mouse for analysis. Images and videos were acquired using a multiphoton microscope (Leica SP5), equipped with a 20× water-dipping objective (NA 1.0) and a MaiTai pulsed infrared laser (SpectraPhysics) set to an excitation wavelength of 810 nm. A 512 × 512 pixel image was acquired every 1.5 s for 10 min. Labelling of the vasculature was performed by intravenous administration of 2.5 µg PE-conjugated anti-CD31 (clone 390, eBioscience).

The video files were imported into FIJI (v1.51, NIH) and converted into composite-color images. Adjustments to brightness and contrast were made to individual files to allow optimal visualization and measurements of blood vessels and cells. At least three measurements of the width of blood vessels were taken and averaged, while the length of the vessel was measured along its center, both using the segmented line tool. Neutrophils were considered rolling if they interacted with the blood vessel for a minimum of 100 µm. Neutrophils were considered adherent if they interacted with the vessel wall for 30 s or more. The number of rolling and adherent/intravascular neutrophils was normalized to the vessel surface area and time of recording. The number of extravascular neutrophils was normalized to the area of colon that was visible in the field of view. The measurements from all fields of view from one mouse were averaged with the final data representing neutrophil numbers from one mouse.

### RNA Isolation and qRT-PCR

Total RNA from colon tissue was extracted using TRI-reagent (Sigma) as per the manufacturer’s protocol. Since DSS has been shown to disrupt the activity of reverse transcriptase, the precipitated RNA samples were then purified of DSS through a lithium chloride purification step (26) and treated with DNase (Invitrogen). The purified RNA was reverse transcribed into cDNA using SuperScript III synthesis system (Invitrogen). Quantitative PCR was performed using Power SYBR Green PCR Master Mix (Applied Biosystems), targeting expression of *18S*, *Cxcl1*, *Cxcl2*, *Icam1*, *Il1b*, *Il10*, *Sele*, *Selp*, *Tgfb*, *Tnfa*, and *Vcam1* (primer sequences can be found at **Supplemental Table 2**). All samples were run in triplicate and normalized to *18S*. Expression of each gene was expressed as fold change relative to mice fed control (CON) diet, with or without DSS.

### Quantification of CXCL2 Protein Levels

Mouse colons were weighed and homogenized in 500 µl of PBS containing cOmplete, EDTA-free Protease Inhibitor Cocktail (Sigma). Colon homogenates were centrifuged at 6,000 *g* for 10 min at 4°C and the supernatant was collected. The presence of chemokines were determined using a mouse CXCL2 ELISA kit (R&D Systems, cat. no. DY452-05) according to manufacturer’s protocol. Briefly, a 96-well ELISA plate (Thermo-Fischer Scientific) was coated with 100 µl of capture antibody (2 µg/ml) and incubated overnight. Plates were washed three times in 0.05% PBS-Tween and blocked with 200 µl of 10% BSA in PBS for 1 h. Plates were washed and standards and colon supernatants were added and incubated for 2 h. Following washing, 100 µl of 75 ng/ml detection antibody was added and incubated for 2 h. Washing was repeated and 100 µl of Streptavidin-HRP was added and incubated for 20 min in the dark. To develop, plates were washed and tetramethylbenzidine (TMB) substrate was added where plates were allowed to develop for 20 min in the dark. To stop the reaction, 50 µl of 1M H2SO4 was added to all wells. A plate reader set at 450 nm was used to measure absorbance in order to generate a standard curve for interpolation of samples.

### Myeloperoxidase (MPO) Assay

Mouse colons were weighed before performing the MPO assay. Briefly, phosphate buffer A was made by dissolving 6.8 g of monobasic potassium phosphate in 1 L distilled water, and phosphate buffer B was made by dissolving 8.7 g of dibasic potassium phosphate in 1 L distilled water. MPO buffer was made by adding phosphate buffer B was to phosphate buffer A until pH 6.0 was reached. Hexadecyltrimethylammonium bromide (HTAB) buffer by adding 5 g HTAB into 1 L of MPO buffer. O-dianisidine solution (ODS) was made fresh on the day by mixing 16.7 mg O-dianisidine HCl in 10 mL MPO buffer, 50 µl of 1% hydrogen peroxide, and 90 mL of distilled water. Tissue samples were homogenized in HTAB buffer at a concentration of 50 mg tissue per mL of HTAB buffer. 1 mL of the homogenate was centrifuged at 5,000 *g* for 4 min. 7 µl of the supernatant was mixed with 200 µl of ODS in a 96 well plate, and absorbance was measured at 405 nm.

### Flow Cytometry of Leukocytes From Colon and Blood

Isolated colons were washed in phosphate buffered saline (PBS) to remove fecal matter, then cut into small pieces and washed in Hank’s balanced salt solution (HBSS, Life Technologies). These samples were incubated in HBSS with 10% fetal calf serum (FCS, Life Technologies) and 5 mM EDTA, then digested in 0.5 mg/mL collagenase D for 90 min and passed through a 70 µm mesh. The cells were resuspended in 40% isotonic Percoll (GE Healthcare) and layered over 80% isotonic Percoll. Gradients were resolved by centrifugation for 20 min at 1,000 *g* with no brake, after which leukocytes were collected from the interface. For isolation of blood leukocytes, cardiac puncture was performed using heparinized syringes. Three hundred microliters of blood were then used for subsequent processing. For all isolated samples, red blood cells were lysed for 1 min, and leukocytes were filtered through a 40 µm mesh.

Cell viability was determined using 7-Aminoactinomycin D (7-AAD), and leukocyte populations and surface CXCR2 expression enumerated using fluorochrome-conjugated monoclonal anti-mouse antibodies against CD45 (30-F11, eBioscience), CD3 (145-2C11, BD Biosciences), CD11b (M1/70, Biolegend), Ly6C (AL-21, BD Biosciences), Ly6G (1A8, Biolegend), and CXCR2 (SA044G4, Biolegend). Cell counts were measured using fluorescent microbeads (BD Biosciences). Fc receptor blocker (CD16/32, clone 2.4G2, BD Biosciences) was used to minimize non-specific binding of antibodies. Cells were analysed on a BD Fortessa X (BD Biosciences) and data analysed using FlowJo (v10.0.7, Tree Star Inc). Populations were defined as live 7-AAD^-^CD45^+^ leukocytes, with further separation into CD3^+^ T cells, CD11b^+^Ly6C^+^Ly6G^-^ monocytes, and CD11b^+^Ly6G^+^Ly6C^-^ neutrophils, unless otherwise noted.

### Statistical Analysis

Student’s *t*-test was used to compare the mean between two groups, except for gene expression data, where the Mann-Whitney’s *U*-test was performed. Two-way ANOVA with matched time points was used to compare the differences between two groups over time. Adonis analysis was performed on PCA analysis of microbiota at the OTU level. All data are represented as mean ± SEM, with individual dots representing each animal, where possible. Comparisons were considered significant if the p-value was < 0.05.

## Results

### Diet Lacking Fiber Alters the Gut Microbial Composition From Day 3 of Diet Feeding

**Figure 1:**
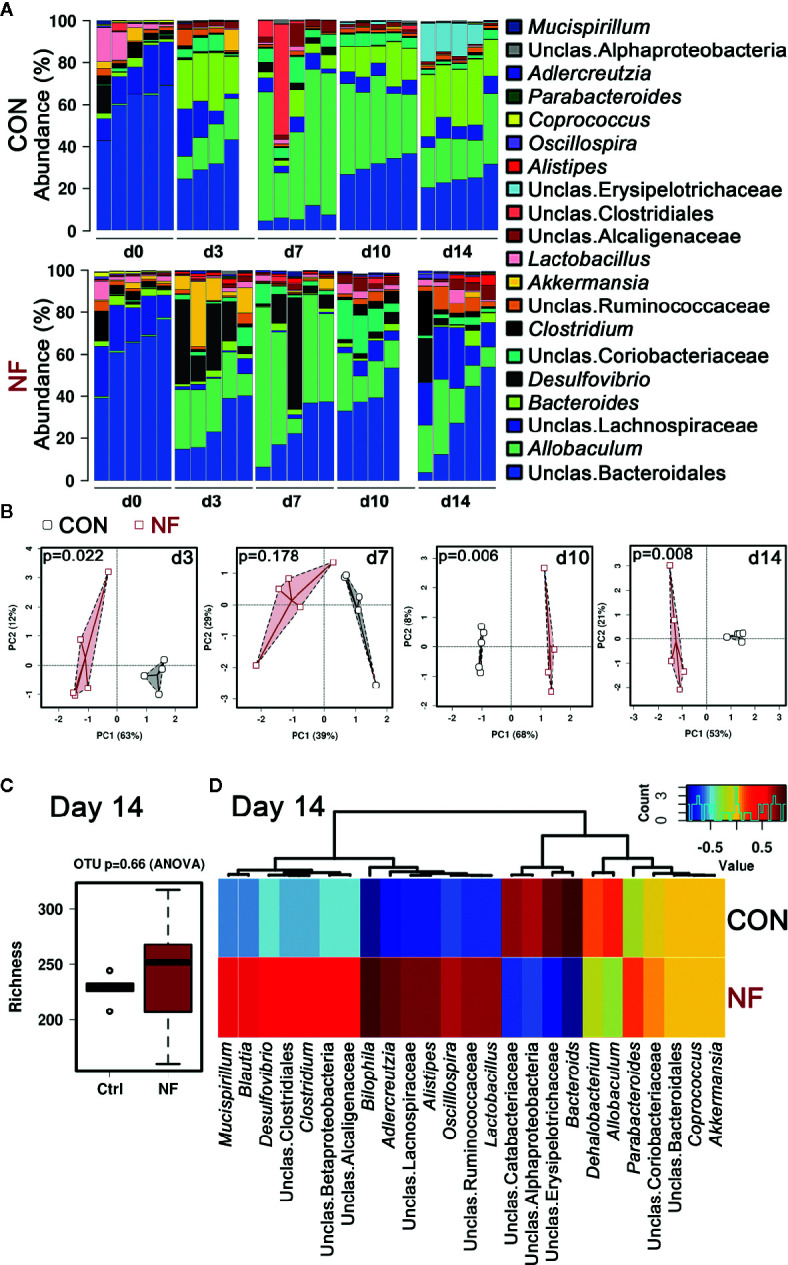
No-fiber diet feeding modifies gut microbiota. WT mice were fed either a control (CON) or no fiber (NF) diet for 14 days. At days 0, 3, 7, 10, or 14 of diet, fecal samples were collected and 16S rRNA sequencing performed. **(A)** The abundance (%) of the top 20 bacteria at the genera level. **(B)** Principle coefficient analysis (PCA) of CON and NF groups at the OTU level, and Adonis UniFrac was performed. At day 14, the diversity of the gut microbial composition was quantified by analysis of richness **(C)** and Pearson’s correlation at the genera level were performed **(D)**. Dots represent individual mice, N ≥ 4 per time point per diet group.

Dietary fiber intake has been shown to play a significant role in modulating the composition of the gut microbiota. Therefore, we examined the impact of a diet lacking fiber on gut microbiota composition over a period of 2 weeks. Age-matched wildtype (WT) littermates were randomly assigned to either control (CON) or no-fiber (NF) diet, and then the fecal microbiota compositions were assessed. It is noteworthy that all the mice consumed and were maintained on a standard chow diet (denoted as Day 0) since weaning, and there was comparable microbiota composition between the experimental groups at this time point (**Supplemental Figure 1A**) with the top 20 most abundant genera in their fecal microbiota being mostly *Bacteroidiales* and *Lachnospiraceae* (**Figure 1A**). After changing from the standard chow to experimental diets, the microbiota composition in both groups were markedly altered from as early as day 3 post-diet (**Figure 1A**). In mice changed to CON experimental diet, there was a decrease in the abundance of *Bacteroidiales* and *Lachnospiraceae*, and an increase in the abundance of predominantly *Bacteroides* and *Allobaculum* (**Figure 1A**). NF diet had a differential effect on the abundance of bacteria when compared to CON diet, with the microbiota in NF-fed mice composed of higher abundance of *Desulfovibrio*, *Akkermansia*, and *Alistipes*, and a marked reduction in the abundance of *Bacteroides* (**Figure 1A**). The proportion of the microbiota that is contributed by each genus was also seen to fluctuate between each time point, possibly reflecting on the constant changing nature of the gut microbiota (**Figure 1A**). Principal component analysis (PCA) of the overall composition at the operational taxonomic unit (OTU) level between CON and NF groups demonstrated significant differences at days 3 (*P* = 0.022), 10 (*P* = 0.006), and 14 (*P* = 0.008; **Figure 1B**).

After 14 days of diet, the animals on control or no-fibre diet gained comparable amount of weight over the 2-week period, thus it is highly likely that these mice had similar food intake, with fiber content as the only difference (**Supplemental Figure 1B**). Moreover, we observed no significant difference in the number of bacterial species in the fecal samples between CON and NF groups, as measured by richness (*P* = 0.66 by ANOVA, **Figure 1C**). However, there was a clear distinction between the composition of the gut microbiota in mice fed CON diet and those fed NF diet at day 14. At the genera level, mice fed a CON diet had high abundance of *Bacteroides*, and lower abundance of *Bilophila*, *Adlercreutzia*, and *Alistipes*, whereas mice fed NF diet harbored *Adlercreutzia*, *Alistipes*, *Bilophila*, *Blautia, Clostridium*, *Desulfovibrio, Mucispirillum*, and *Oscillospira*, and reduced abundance of *Bacteroides* (**Figure 1D**). Taken together, a diet lacking fiber resulted in an altered Dmicrobial composition that was seen from as early as day three. Moreover, these results indicate that the changes in gut microbiota composition induced by removal of fiber are not static, but are dynamic and continue to evolve over time.

### Diet Lacking Fiber Increases *Colonic* Cxcl2 Expression and Neutrophil-Endothelial Cell Interactions

**Figure 2:**
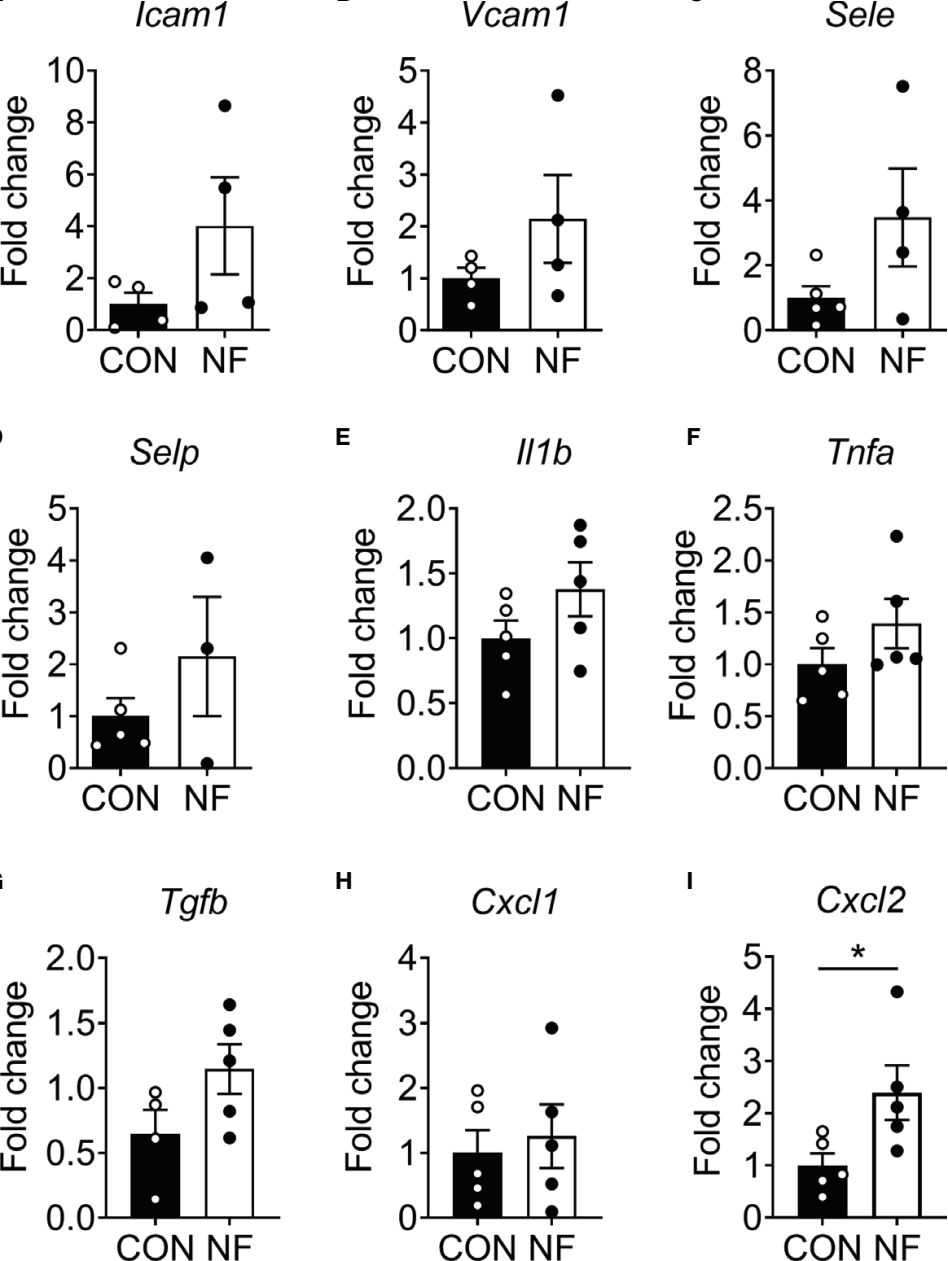
Cytokine gene expression profile in the colon following no-fibre feeding. WT mice were fed CON or NF diet for 14 days. At day 14, the expressions of **(A)** *Icam1*, **(B)** *Vcam1*, **(C)** *Sele*, **(D)** *Selp*, **(E)** *Il1b*, **(F)** *Tnfa*, **(G)** *Tgfb*, **(H)** *Cxcl1* and **(I)** *Cxcl2* in the colon of WT mice were quantified relative to mice fed CON diet. Data displayed as mean ± SEM. Dots represent individual mice, N ≥ 4 per group, *P < 0.05 with the Mann-Whitney *U*-test.

To examine the effect of a diet lacking fiber and its associated changes in the gut microbiota on the host colonic microenvironment, we assessed the expression of various inflammatory genes associated with leukocyte recruitment in the colon, including adhesion molecules (*Icam1, Vcam1, Sele*, and *Selp*), inflammatory cytokines (*Il1b, Tnfa*, and *Tgfb*) and chemotactic cytokines (*Cxcl1* and *Cxcl2*). Compared to mice fed a CON diet, we found no changes in the expression of a panel of genes associated with leukocyte adhesion or inflammation in mice fed NF diet at day 14 of feeding (**Figures 2A–H**). However, the gene expression of a neutrophil-attracting chemokine *Cxcl2* was significantly higher in mice fed a NF diet compared to CON diet fed mice after 14 days of feeding on experimental diet (**Figure 2I**), though protein levels remained unchanged (**Supplemental Figure 2**).

**Figure 3:**
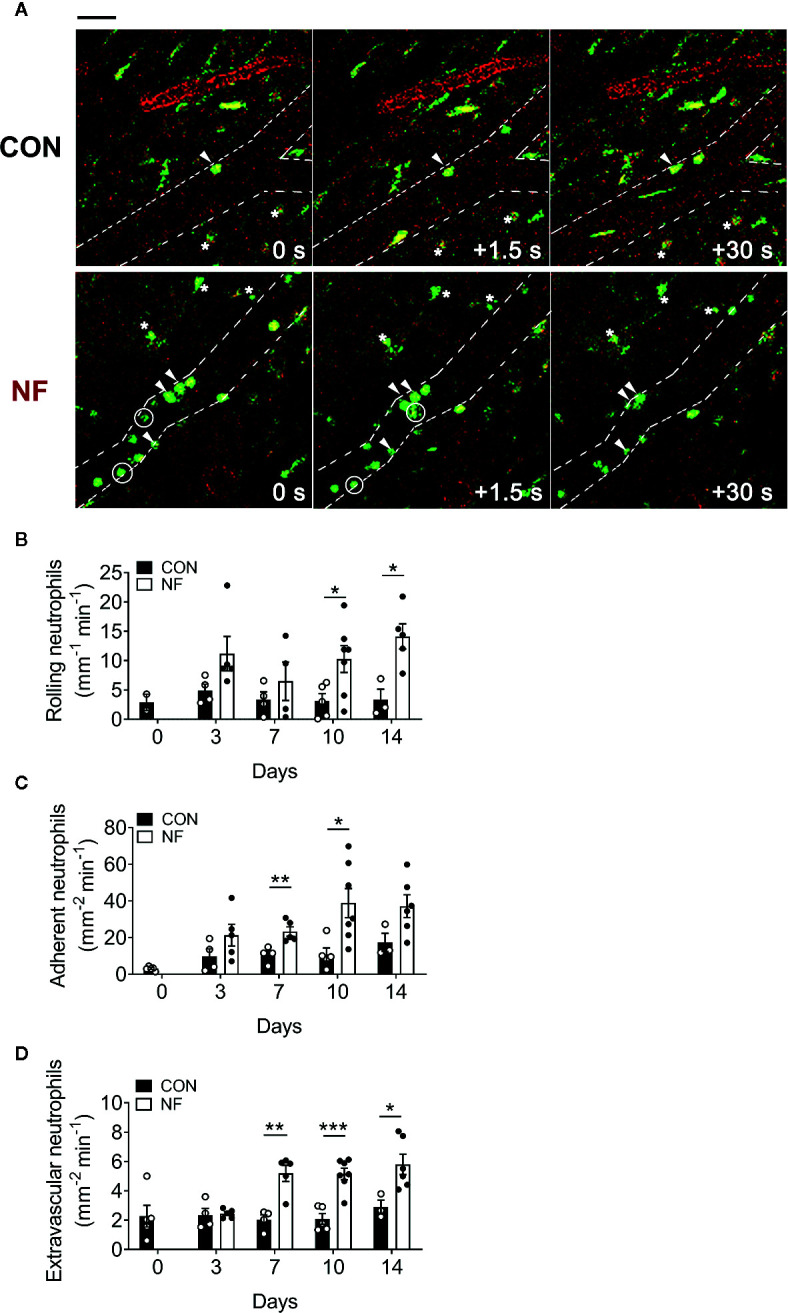
No-fiber diet promotes the recruitment of neutrophils to the colon. *LysM^eGFP^* mice were fed CON or NF diet for 14 days. At days 0, 3, 7, 10, and 14, *LysM^eGFP^* mice were anesthetized, injected with PE-conjugated anti-CD31 to label the endothelium, and intravital microscopy of the colon was performed. **(A)** Representative image sequences taken at day 14 of CON or NF diet feeding, showing neutrophils that are rolling (circled), adherent (arrowhead), and extravascular (asterisk). Scale bar = 50 μm. The number of **(B)** rolling, **(C)** adherent, and **(D)** extravascular neutrophils were enumerated. Data displayed as mean ± SEM. Dots represent individual mice, N ≥ 3 per group per timepoint. Student’s *t*-test was performed, *P < 0.05, **P < 0.01, ***P < 0.001.

Next, we investigated if the elevated *Cxcl2* gene expression in the colon following NF diet is associated with increased neutrophil interactions with the colonic microvasculature and recruitment into the colon. Utilizing colon intravital imaging of neutrophil reporter (*LysM^eGFP^*) mice at days 0 (prior to experimental diets), 3, 7, 10, and 14 post-diet, temporal changes in neutrophil-endothelial cell interactions in the colonic vasculature were profiled. In mice fed a CON diet, we observed minimal alterations in the numbers of rolling, adherent, and extravascular neutrophils throughout the 14 days of diet (representative images taken at day 14 are shown in **Figure 3A**, quantitative data shown in **Figures 3B–D**), suggesting the change from standard chow (day 0) to CON diet induced minimal impact on neutrophil-endothelial cell interactions. In comparison, mice fed a NF diet showed increasing numbers of rolling neutrophils from day 7 (**Figure 3B**), significantly greater adherent neutrophils at days 7 and 10 (**Figure 3C**), and markedly elevated extravascular neutrophils from day 7 (**Figure 3D**). Therefore, our results suggest that a gut microbiota shaped by a diet deficient in fiber is associated with an increased colonic expression of *Cxcl2* and an elevation of neutrophil-endothelial cell interactions at the colonic microvasculature.

### Mice Fed a NF Diet Are Highly Susceptible to DSS-Induced Colitis

**Figure 4:**
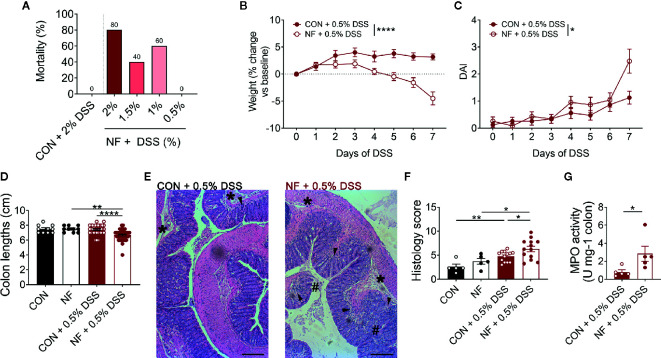
No-fiber diet exacerbates DSS-induced colitis. WT mice were fed either a control (CON) or no fibre (NF) diet for 14 days prior to and during DSS administration. **(A)** Mortality rate (%) at day 7 following 0.5, 1, 1.5, or 2% DSS added to drinking water (N = 5 per group). During 0.5% DSS-induced colitis, there was daily monitoring of **(B)** weight (N = 23 per group) and **(C)** DAI (N = 23 per group). **(D)** At day 7 of 0.5% DSS-induced colitis, mice were culled, and colon length was measured (N ≥ 10 per group). **(E)** The colon was fixed, and histological sections were stained using hematoxylin and eosin. Scale bar = 400 µm. **(F)** Tissues were blindly scored for parameters assessing tissue involvement, inflammation (arrowhead), edema (*), and epithelial damage (#) (N ≥ 5 per group). **(G)** In separate experiments, MPO activity of isolated colons were quantified relative to mice fed a CON diet and administered with DSS (N ≥ 5 per group). Data displayed as mean ± SEM. Two-way ANOVA was performed for all data across all time point for **(B, C)**, Student’s *t*-test was performed for **(D, F, G)**, *P < 0.05, **P < 0.01, ****P < 0.0001.

While a diet lacking fiber results in dysbiosis, upregulation of colonic *Cxcl2* gene expression and increased neutrophil-endothelial interactions, it is notable that a fiber-deficient diet was insufficient to induce detectable pathology in the colon. Specifically, 2 weeks feeding of NF diet alone did not result in obvious macroscopic pathology such as colonic tissue edema, colon length shortening or occult. Therefore, we hypothesized that a diet lacking fiber predisposes mice to increased susceptibility to colonic inflammation, such as DSS-induced colitis. To test this hypothesis, mice were randomly assigned to either the CON or NF diet for two weeks prior to, and during, the 7 days of DSS-induced colitis. We initially administered DSS at a concentration of 2% in drinking water, which we have previously shown to elicit moderate disease in mice on the CON diet (24). However, mice fed the NF diet and administered 2% DSS quickly developed severe disease and had to be euthanized. By day 7, there was 80% mortality in mice fed the NF diet and 2% DSS, 40% mortality in mice administered 1.5% DSS, and 60% mortality in mice administered 1% DSS (**Figure 4A**). It was only with a further reduction to 0.5% DSS that there was no mortality in mice fed a NF diet (**Figure 4A**). Therefore, 0.5% DSS was used for the remainder of this study. More importantly, these results provide evidence of the fatal effects of a diet lacking fiber in an experimental model of colitis.

With administration of 0.5% DSS in drinking water, mice fed a CON diet did not lose any weight throughout the 7 days of DSS administration (**Figure 4B**), displayed minimal DAI (**Figure 4C**), and showed no shortening of the colon at the experimental endpoint as a readout of colonic pathology (**Figure 4D**). In contrast, mice fed the NF diet and then exposed to 0.5% DSS showed significant weight loss (**Figure 4B**), increased DAI (**Figure 4C**), and significantly shortened colons (**Figure 4D**). When assessed for tissue inflammation and damage at the experimental endpoint, 0.5% DSS administration resulted in increased leukocyte infiltration, edema, and damage to the epithelium in both mice fed CON or NF diet when compared to their respective water control groups (representative image **Figure 4E**; quantified in **Figure 4F**). Interestingly, inflammation was particularly exacerbated in mice fed the NF diet post-DSS when compared to mice fed the CON diet (**Figure 4F**). Despite this, we did not detect evidence for gut permeability in either experimental groups following 7-day of 0.5% DSS-induced colitis. MPO activity in the whole colon was also quantified as a marker of inflammation and neutrophil recruitment. Following 0.5% DSS administration, mice fed the NF diet had approximately a three-fold increase in MPO activity level compared to mice fed the CON diet (**Figure 4G**).

Furthermore, we performed additional correlations to draw stronger links between bacterial strains differentially represented in the gut microbiota of mice fed on a control versus no-fibre diet. The results showed statistically significant negative correlation of the presence and abundance of Verrucomicrobia at the phylum level (**Supplemental Table 3**) and *Akkermansia* at the genus level (**Supplemental Table 4**) with the DAI following DSS-induced colitis in the NF feeding cohort (**Supplemental Figure 3**). Taken together, these results show that mice fed a diet lacking fiber are highly susceptible to DSS-induced intestinal inflammation.

### No-Fiber Diet *Elevates* Expression of Inflammatory Genes and Neutrophil Adhesion in the Colon Post-DSS

**Figure 5:**
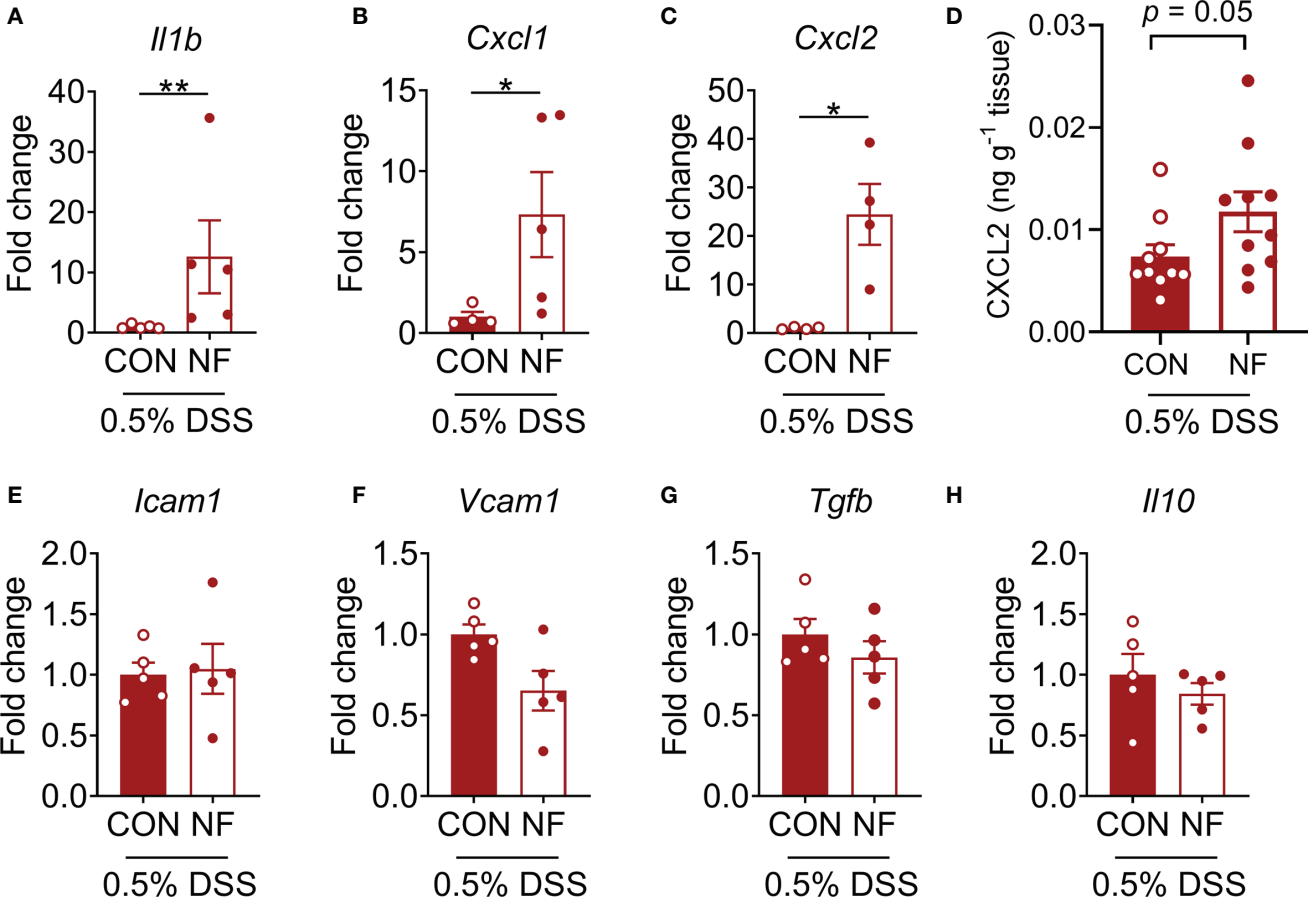
Colonic cytokine and neutrophil recruitment profiles. WT mice were fed either a control (CON) or no fiber (NF) diet for 14 days prior to and during 7 days of 0.5% DSS-induced colitis. At experimental endpoint, RNA was isolated from the colon, and expression of **(A)** *Il1b*, **(B)** *Cxcl1*, **(C)** *Cxcl2* **(E)** *Icam1*, **(F)** *Vcam1*, **(G)** *Tgfb* and **(H)** *Il10* were assessed. Data was normalized to CON DSS group, presented as mean ± SEM. N ≥ 4 per group, *P < 0.05, **P < 0.01 with the Mann-Whitney *U*-test. At experimental endpoint, protein was isolated from the colon and levels of CXCL2 **(D)** was assessed. Data was presented as mean ± SEM. N ≥ 10 per group, *P*-value with the Mann-Whitney *U*-test.

**Figure 6:**
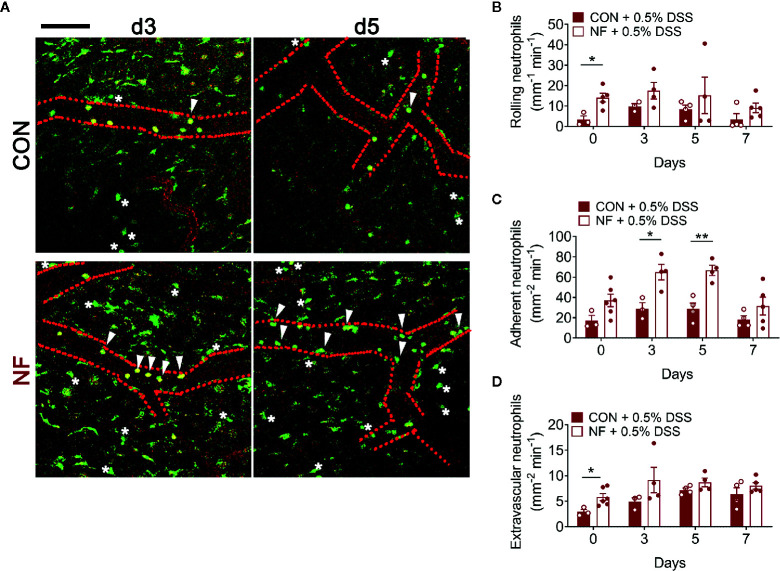
No-fiber feeding exacerbates neutrophil-endothelial interactions post-DSS. WT mice were fed either a control (CON) or no fiber (NF) diet for 14 days prior to and during 7 days of 0.5% DSS-induced colitis. **(A)** At days 0, 3, 5, and 7 post-DSS, mice were anesthetized, injected with PE-conjugated anti-CD31 to label the endothelium, and intravital microscopy of the colon was performed. Scale bar = 100 µm. The number of **(B)** rolling, **(C)** adherent, and **(D)** extravascular neutrophils were enumerated. Data displayed as mean ± SEM. Dots represent individual mice, N ≥ 3 per group per timepoint, *P < 0.05, **P < 0.01 with Student’s *t*-test.

To assess whether a NF diet alters the colonic microenvironment post-DSS, the expression of various inflammatory genes in the colon was assessed. Compared to mice fed the CON diet, 0.5% DSS challenge following NF feeding significantly increased the gene expression of *Il1b*, *Cxcl1* and *Cxcl2* post-DSS (**Figures 5A–C**), although the expression of adhesion molecules and anti-inflammatory cytokines did not differ between the groups (**Figures 5E–H**). Moreover, the protein levels of CXCL2 is elevated in NF-fed mice after DSS-colitis compared to their control counterparts (**Figure 5D**). In addition, we examined the immune composition in the colon at experimental endpoint and found the majority of leukocytes that infiltrated the colonic tissue following this experimental model of colitis is attributed to neutrophils (**Supplemental Figure 4**). In light of these observations, intravital microscopy of the colon was performed at days 0, 3, 5, and 7 following 0.5% DSS administration to investigate whether NF diet-induced alteration in the colonic microenvironment increased neutrophil-endothelial cell interactions post-DSS. Minimal changes were observed in the number of rolling, adherent and extravascular neutrophils in mice fed CON diet during the period of DSS administration (representative images **Figure 6A**; quantified in **Figures 6B–D**). In contrast, mice fed the NF diet had significantly increased numbers of adherent neutrophils at days 3 and 5 when compared to those fed the CON diet (**Figure 6C**). However, no significant differences were observed in the numbers of rolling (**Figure 6B**) and extravascular neutrophils (**Figure 6D**). Together, the findings indicate that the colonic microenvironment established by NF feeding is pro-inflammatory and chemotactic, allowing for elevated neutrophil adhesion to the colon microvasculature following induction of a very mild form of colitis.

### Supplementation of NF Diet With Acetate Prevents DSS-Induced Colitis

**Figure 7:**
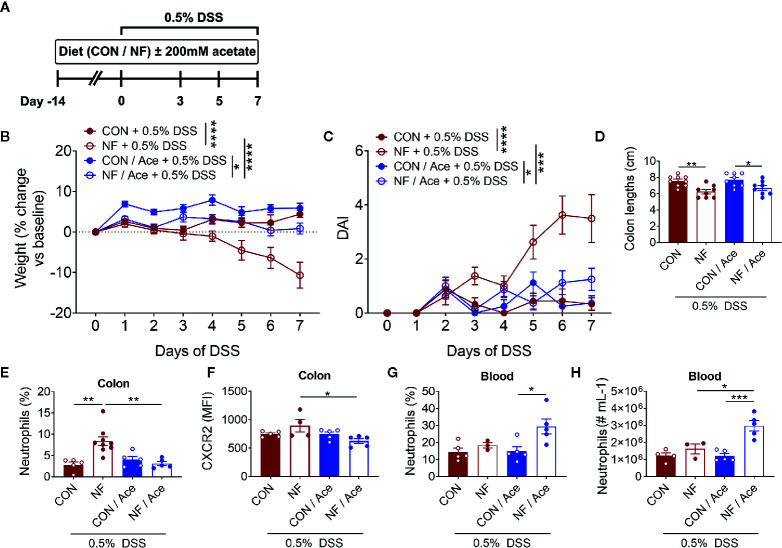
Effect of supplementing acetate with NF diet on development of DSS-induced colitis. **(A)** WT mice were supplemented with 200 mM acetate in drinking water in addition to either a control (CON) or no fiber (NF) diet for 14 days prior to and during 0.5% DSS-induced colitis. There was daily monitoring of **(B)** weight (N ≥ 8 per group) and **(C)** DAI (N ≥ 8 per group) during 0.5% DSS-induced colitis. **(D)** At day 7 of 0.5% DSS-induced colitis, mice were culled, and colon length was measured (N ≥ 8 per group). The colon was collected, and neutrophils were quantitated by flow cytometry using fluorescent counting beads. The proportion **(E)** and expression of CXCR2 **(F)** on neutrophils in the colon and blood **(G, H)** were enumerated. Data displayed as mean ± SEM, N ≥ 3 per group. Two-way ANOVA was performed for all data across all time point for **(B, C)**. Student’s *t*-test was performed for **(D–H)**, *P < 0.05, **P < 0.01, ***P < 0.001, ****P < 0.0001.

We next assessed whether addition of the SCFA acetate to the NF diet could shift to the colonic microenvironment to one that is anti-inflammatory, and thereby protect against experimental colitis. In these sets of experiments, acetate was supplemented in the drinking water for 14 days during the period of the experimental diets leading up to induction of the 0.5% DSS colitis model and throughout the 7-day duration of the model (**Figure 7A**). As expected, mice fed the NF diet showed significant weight loss and increased DAI when compared to mice fed a CON diet (**Figures 7B, C** **)**. Acetate supplementation in mice on NF diet prevented the weight loss and rise in DAI when compared to mice on NF diet without acetate (**Figures 7B, C** **)**. However, acetate supplementation had no effect on colon lengths in mice fed either CON or NF diets (**Figure 7D**).

We then examined whether acetate supplementation altered colonic neutrophil recruitment following 0.5% DSS. NF diet induced a significant increase in the proportion of neutrophils at day 7 of 0.5% DSS, and acetate supplementation significantly reduced the proportion of neutrophils (**Figure 7E**, **Supplemental Figure 5A**). The chemokine receptor CXCR2, which binds CXCL1, and CXCL2, has a major role in chemokine-mediated recruitment of neutrophils. Therefore, we proposed that the lowered proportion of colonic neutrophils in mice fed a NF diet and supplemented with acetate is mediated by hindered chemoattraction to the colon. We tested this hypothesis by flow cytometric analysis of the median fluorescent intensity (MFI) of CXCR2 as a measure of its expression on neutrophils. We found that at the endpoint of DSS-induced colitis, there were no differences in CXCR2 expression on a per neutrophil basis between mice fed a CON or NF diet (**Figure 7F**). However, in mice fed a NF diet, supplementation of acetate significantly reduced CXCR2 expression on neutrophils (**Figure 7F**, **Supplemental Figure 5B**). Therefore, supplementation of acetate prevented the accumulation of colonic neutrophils and lowered their CXCR2 expression in mice fed a NF diet post-DSS. Furthermore, we tested the hypothesis that acetate-induced reduction of CXCR2 expression may also affect neutrophils in the circulation, thereby promoting their retention in the circulation and restricting them from accumulating in the colon. Indeed, the proportion and number of neutrophils in the blood were markedly elevated in NF-fed mice treated with acetate (**Figures 7G, H** **)**. These findings suggest the protective effect of acetate supplementation in the absence of dietary fibre following experimental colitis is accompanied by reduced CXCR2 expression and infiltration of colonic neutrophils, possibly associated with increased retention of these cells in the peripheral circulation.

## Discussion

Elevated consumption of dietary fiber modulates the gut microbiota and protects against the development of inflammatory conditions such as colitis, asthma, and hypertension (7, 8, 11, 27). However, the effect of a no-fiber diet on leukocyte-endoethial cell interactions at a basal state, and in the pathogenesis of colitis remains to be fully elucidated. We show that reduced fiber intake modifies gut microbiota composition in as little as three days and that this response continues to evolve for at least 2 weeks. In addition, the gut microbiota resulting from a diet that lacks fiber is completely different to that found in mice fed a normal control diet. Although a no-fiber diet was associated with increased neutrophil-endothelial cell interactions in the colon from day 7 and upregulated colonic *Cxcl2* expression at day 14, it did not induce observable colonic tissue damage or colitis-related pathology. However, consumption of a fiber-deficient diet greatly increased the susceptibility of wildtype mice to 0.5% DSS-induced colitis. This was accompanied by an increase in neutrophil-endothelial interactions in the colonic vasculature, elevated expression of *Il1b*, *Cxcl1*, and *Cxcl2* in the colon, and exacerbated MPO levels. Interestingly, our findings suggest acetate supplementation to no-fiber feeding have systemic anti-inflammatory capacities (11) and shift the colonic environment to one that consists of reduced neutrophil expression of CXCR2 and infiltration, potentially contributing to the observed protection against DSS-induced colitis.

In concordance to prior studies, a change in diet altered the gut microbiota composition within three days (28). However, the composition of the microbiota continued to evolve throughout the 14 days of diet, highlighting the complex and dynamic interplays between microbial communities. It is important to note that variability of the microbiota composition is also present within the same group of mice. This confirms the accepted notion that a multitude of factors including founder effects during initial microbiota establishment, food intake, physical activity, or physiological stress also contribute to inter-animal variability in colonic microbial communities (29). When mice were fed a fiber-deficient diet for 14 days, there was increased abundances of *Bilophila* and *Alistipes*, and decreased proportion of *Bacteroides*, which is consistent with previously published literature (7, 10). Furthermore, we also observed higher abundances of *Adlercreutzia*, *Desulfovibrio*, and *Mucispirillum*. All of these changes have been associated with a Western diet, inflammation, and inflammatory bowel disease (7, 10, 24, 30–32). Interestingly, these gradual changes towards an “inflammatory” gut microbiota correlated with increased neutrophil–endothelial interactions in the colon. While very unlikely, it is currently unknown whether colonization with a single strain of bacteria has the ability to modulate neutrophil–endothelial interactions in the colon. Instead, our findings indicate that continuous consumption of a no-fiber diet enriches for multiple strains of bacteria that are associated with inflammation and may indeed alter gut immune homeostasis, potentially predisposing the host to inflammatory diseases. In addition, our results suggest that future studies should increasingly examine the temporal development of microbial communities, instead of solely correlating disease to a single snapshot of the microbiota composition at one time.

A fiber-deficient diet and the resultant dysbiotic microbiota can play a role in amplifying the detrimental and inflammatory effects of DSS-induced colitis. The experimental no-fibre diet used in this project is modelling an extreme type of Western diet where there is a complete lack of fibre content. We have previously shown mice on a low-fibre diet also displayed detrimental effects following DSS-induced colitis, however it was less damaging compared to no-fibre diet in the same study (7). Evidence indicates that DSS-induced intestinal inflammation allows translocation of gut bacteria, and this has been associated with an increased inflammatory response and worsened outcomes of colitis (33). Consistent with prior research, we show that mice fed on a NF diet for 2 weeks demonstrated significant weight loss and worsened colitis pathology compared to mice that were on a fiber-sufficient control diet (7). This could be a direct sensing and response of immune cells to the dysbiotic microbiota as a result of a fiber-deficient diet. Certainly, some bacteria enriched by a no-fiber feeding could degrade mucin as a method of entering the colonic mucosa (34, 35), and together with bacterial ligands such as lipopolysaccharide (LPS), could induce a microenvironment that promotes the recruitment of leukocytes and elicits inflammatory responses. Furthermore, our correlative analysis demonstrated strong links between bacterial strains differentially represented in the gut microbiota of mice fed on a control (CON) versus no-fibre (NF) diet. Specifically, we found negative correlation of the presence and abundance of Verrucomicrobia at the phylum level and *Akkermansia* at the genus level with the disease activity index (DAI) in the NF feeding cohort following DSS-induced colitis. Indeed, the findings of our study suggest a fiber-deficient diet alters the microbiota and colonic environment to one that is pro-inflammatory, resulting in elevated neutrophil recruitment that exacerbate colitis pathology.

In previous studies of colonic inflammation, butyrate has been the predominant SCFA examined, due to the utilisation of this SCFA by colonic epithelial cells as an energy source for maintenance of gut homeostasis (36). However, in this study, we elected to assess the effect of acetate in modulating DSS-induced colitis because, following fiber-feeding, acetate is the SCFA with the highest concentration in both the colonic lumen and the circulation (37). Indeed, we have previously demonstrated the ability of acetate to modulate inflammation-associated leukocyte recruitment in the intestine (38). Moreover, protective effects of acetate and other SCFAs have been reported to be conferred through their ability to directly induce regulatory T cells (Treg) and increase their functional capacity (7, 8, 11, 39, 40), and through shifts of the gut microbiota similar to those observed following a high fiber diet (27). Due to this, we performed experiments on mice deficient of G-protein coupled receptor 43 (*Gpr43^-/-^*) which is thought to be a functional receptor for the protective effects of acetate. However, the findings demonstrated no difference in the degree of disease severity between wildtype and *Gpr43^-/-^* mice. There are a couple of possible explanation for this. Firstly, due to the damaging and premature deaths of animals when they were put on a no-fibre diet for 2 weeks followed by 1–2% DSS, we titrated our model for this study to only 0.5% DSS. As such, the subtle change in pathophysiology following this mild model of 0.5% DSS-induced colitis may have been missed. Secondly, we have previously shown metabolite-sensing receptors GPR43 and GPR109A in combination facilitate dietary fibre-induced gut homeostasis through regulation of the inflammasome (7). Therefore, the removal of only one of the receptors may not be sufficient to demonstrate the reduced protective effects of acetate supplementation. Furthermore, we have previously shown acetate-induced HDAC inhibition in Treg elevated their functional capacity following maternal acetate supplementation in an offspring experimental asthma model (8). However, we found no changes in Treg in our experimental colitis model. In addition, SCFAs also have various other mechanisms of action, such as inducing mucosal IgA and increasing intestinal barrier integrity, as has been extensively reviewed by Tan et al. (11). Clearly, SCFAs are capable of modulating the host inflammatory response *via* mechanisms that affect immune cells systemically or more specifically in the localized site of inflammation. In this study, we showed elevated neutrophil chemoattractant CXCL2 protein expression in the post-DSS colonic tissue of mice fed no-fibre diet, and that supplementation of acetate in the diets of these mice demonstrated significantly reduced expression of the corresponding receptor, CXCR2, on colonic neutrophils and lowered their infiltration into the inflamed colon. These findings suggest that acetate may play a role in modulating the CXCL2-CXCR2 interactions, possibly through retention of neutrophils in the circulation, and effectively prevented the development of experimental colitis.

In this study, we described the temporal profile of colonic neutrophil recruitment during NF feeding and DSS-induced colitis with the use of intravital microscopy. Despite this, our experiments on neutrophil recruitment only examined microvasculature near the surface of the serosal side of the colon, due to limitations in the microscopy technique applied, which could potentially undermine localized hotspots of extreme neutrophil infiltration. In addition, although the use of reporter mice allows visualisation of cells within the tissue, it is impractical to differentiate between neutrophils and other myeloid cells *in vivo* using *LysM^eGFP^* mice. This prompted the use of flow cytometry to acquire a holistic view of colonic leukocyte populations. Recent technical advancements mean newer multiphoton fluorescent microscopes have the capacity for deeper tissue imaging, allowing us to integrate leukocyte recruitment and infiltration into the lamina propria or mucosa. Another method is to perform imaging from the luminal side of the colon, which would enable imaging of leukocytes on the mucosa. However, this would expose a normally anaerobic environment and not ideal for *in vivo* studies.

In summary, our results demonstrate the detrimental effect of a diet lacking fiber, in both health and disease. At as early as day 3, a diet that lacks fiber results in the enrichment of bacteria that have previously been associated with inflammation and inflammatory diseases, potentially increasing the inflammatory state of the colonic environment. This higher basal inflammatory state predisposes the host to worsened pathology after DSS-induced colitis. Supplementation of acetate normally produced during fiber-feeding has the ability to modulate neutrophil recruitment and infiltration, and consequently improve disease outcomes following experimental colitis.

## Data Availability Statement

The datasets presented in this study can be found in online repositories. The names of the repository/repositories and accession number(s) can be found in the article/**Supplementary Material**.

## Ethics Statement

The animal study was reviewed and approved by MMC B Animal Ethics Committee.

## Author Contributions

CW, MH, and SS conceived and planned the experiments. SS, with the help of KP, SW, RS, BW, and CW, carried out the experiments and analysis. For gut microbiota analysis, SS contributed to sample preparation, RM and TV performed sequencing, and DS and SS performed analysis and interpretation of the results. RR supplied the anti-CXCR2 antibody. SS and CW wrote the manuscript with input from all authors. All authors contributed to the article and approved the submitted version.

## Funding

This work was supported by CSL Centenary Fellowship (CW). MH is supported by an NHMRC Senior Research Fellowship (1042775). The funding bodies have no role in the design of the study and collection, analysis, and interpretation of data and in writing the manuscript.

## Conflict of Interest

The authors declare that the research was conducted in the absence of any commercial or financial relationships that could be construed as a potential conflict of interest.
