# Supplementary material for: Deficiency of Dietary Fiber Modulates Gut Microbiota Composition, Neutrophil Recruitment and Worsens Experimental Colitis
Source: Front Immunol. 2021 Feb 23;12:619366. doi: 10.3389/fimmu.2021.619366 (PMC7940676; doi:10.3389/fimmu.2021.619366)
Supplement: Supplementary file 1 [file DataSheet_1.docx]

**Deficiency of dietary fiber modulates gut microbiota composition, neutrophil recruitment and worsens experimental colitis**

Sj Shen^1^, Kathryn Prame Kumar^1^, Shu Wen Wen^1^, Raymond Shim^1^, Brooke J. Wanrooy^1^, Dragana Stanley^2^, Robert J. Moore^3, 4^, Thi Thu Hao Van^4^, Remy Robert^5^, Michael J. Hickey^1^ and Connie H. Y. Wong^1,^ *

^1^ Centre for Inflammatory Diseases, Department of Medicine, School of Clinical Sciences at Monash Health, Monash Medical Centre, Monash University, Clayton, Victoria, Australia.

^2^ School of Health Medical and Applied Sciences, Central Queensland University, Rockhampton, Australia;

^3^ Infection and Immunity Program, Monash Biomedicine Discovery Institute and Department of Microbiology, Monash University, Australia;

^4^ School of Science, Royal Melbourne Institute of Technology (RMIT) University, Australia;

^5^ Monash Biomedicine Discovery Institute and Department of Physiology, Monash University, Clayton, Victoria, Australia.

*Address for correspondence: Connie H. Y. Wong, PhD., Centre for Inflammatory Diseases, Department of Medicine, School of Clinical Sciences at Monash Health, Monash Medical Centre, Monash University, Clayton, VIC 3168 Australia; Email: [connie.wong@monash.edu](mailto:connie.wong@monash.edu)

Running title: Dietary fiber and neutrophil recruitment

**Data deposition statement:**

All the sequencing data are submitted to the Metagenomics Analysis Server (MG-RAST) under project ID 433886.

**Supplementary material**

Supplementary Tables: 4

Supplementary Figures: 5

**Supplemental Table 1.** Composition of the experimental diets.

|  | Control (Ctrl) diet | No fibre (NF) diet |
| --- | --- | --- |
| Diet code from Specialty Feeds | SF09-091  (g/kg) | SF09-028  (g/kg) |
| Casein (Acid) | 200 | 200 |
| Sucrose | 100 | 0 |
| Canola Oil | 70 | 70 |
| Cellulose | 50 | 0 |
| Wheat Starch | 399 | 0 |
| Dextrinised Starch | 132 | 0 |
| DL Methionine | 3.0 | 3.0 |
| Calcium Carbonate | 13.1 | 13.1 |
| Sodium Chloride | 2.6 | 2.6 |
| AIN93 Trace Minerals | 1.4 | 1.4 |
| Potassium Citrate | 2.5 | 2.5 |
| Potassium Dihydrogen Phosphate | 6.9 | 6.9 |
| Potassium Sulphate | 1.6 | 1.6 |
| Choline Chloride (75%) | 2.5 | 2.5 |
| AIN93 Vitamins | 15 | 10 |
| Vitamin K Supplement | 0.87 | 0 |
| Dextrose Monohydrate | 0 | 686 |

**Supplemental Table 2.** Primer sequences

| Primer target | | Sequence (5’ – 3’) |
| --- | --- | --- |
| *18S* | F | CTT AGA GGG ACA AGT GGC G |
|  | R | ACG CTG AGC CAG TCA GTG TA |
| *Cxcl1* | F | CCG AAG TCA TAG CCA CAC TCA A |
|  | R | GCA GTC TGT CTT CTT TCT CCG TTA C |
| *Cxcl2* | F | CCA ACC ACC AGG CTA CAG G |
|  | R | GCG TCA CAC TCA AGC TCT G |
| *Icam1* | F | CAC CGT GTA TTC GTT TCC G |
|  | R | TGA GGT CCT TGC CTA CTT GC |
| *Il1b* | F | GTC GCT CAG GGT CAC AAG AA |
|  | R | GTG CTG CCT AAT GTC CCC TT |
| *Il10* | F | GTA GAA GTG ATG CCC CAG GC |
|  | R | AAA TCG ATG ACA GCG CCT CAG |
| *Sele* (E-selectin) | F | CAT GCA AAG CTG TGA CCT GT |
|  | R | TGC AAC GTG AAA CTC TGC TC |
| *Selp* (P-selectin) | F | GTC CAC GGA GAG TTT GGT GT |
|  | R | TGT TAT GCC TTT GCA GGT TG |
| *Tgfb* | F | GAG CCA GAA CGA GAA GTA CCG |
|  | R | CCT CAA GAC GAG CAA TTT CAT CA |
| *Tnfa* | F | ATG AGC ACA GAA AGC ATG ATC CGC |
|  | R | CCA AAG TAG ACC TGC CCG GAC TC |
| *Vcam1* | F | TTT GGA AAC GAC CTT CAT CC |
|  | R | GGG CAA CGT TGA CAT AAA GAG |

**Supplemental Table 3.** Correlations between bacterial strains differentially represented in the gut microbiota of mice fed on a no-fibre diet and Disease Activity Index (DAI) at the phylum level.

| Taxa | P value | R | Mean Abundance |
| --- | --- | --- | --- |
| Verrucomicrobia | 0.0016 | -0.9984 | 0.515 |
| Actinobacteria | 0.072 | -0.928 | 1.638 |
| Deferribacteres | 0.24 | 0.7635 | 0.325 |
| Tenericutes | 0.57 | 0.4343 | 4.285 |
| Firmicutes | 0.73 | 0.2715 | 5.535 |
| Bacteroidetes | 0.74 | -0.2596 | 5.297 |
| Proteobacteria | 0.92 | -0.0814 | 2.995 |

**Supplemental Table 4.** Correlations between bacterial strains differentially represented in the gut microbiota of mice fed on a no-fibre diet and Disease Activity Index (DAI) at the genus level.

| Taxa | P value | R | Mean Abundance |
| --- | --- | --- | --- |
| Akkermansia | 0.0016 | -0.9984 | 0.515 |
| Unclassified.Coriobacteriaceae | 0.16 | -0.8381 | 1.462 |
| Blautia | 0.16 | -0.8442 | 0.112 |
| Mucispirillum | 0.24 | 0.7635 | 0.325 |
| Unclassified.Catabacteriaceae | 0.36 | -0.6427 | 0.242 |
| Unclassified.Alphaproteobacteria | 0.36 | -0.6378 | 0.202 |
| Unclassified.Lachnospiraceae | 0.4 | 0.5986 | 3.978 |
| Bilophila | 0.4 | 0.5995 | 0.53 |
| Unclassified.Betaproteobacteria | 0.47 | -0.5304 | 0.167 |
| Bacteroides | 0.47 | -0.5261 | 1.548 |
| Unclassified.Erysipelotrichaceae | 0.49 | -0.5119 | 0.362 |
| Allobaculum | 0.54 | 0.4587 | 4.247 |
| Unclassified.Clostridiales | 0.56 | 0.4382 | 0.29 |
| Lactobacillus | 0.63 | 0.3713 | 1.242 |
| Parabacteroides | 0.68 | -0.3215 | 0.57 |
| Unclassified.Bacteroidales | 0.77 | -0.2306 | 4.872 |
| Dehalobacterium | 0.8 | 0.2015 | 0.132 |
| Oscillospira | 0.82 | 0.1791 | 1.062 |
| Alistipes | 0.86 | -0.14 | 1.18 |
| Desulfovibrio | 0.88 | -0.1155 | 2.143 |
| Clostridium | 0.89 | -0.1116 | 2.163 |
| Unclassified.Alcaligenaceae | 0.92 | -0.0814 | 1.44 |
| Unclassified.Ruminococcaceae | 0.93 | -0.0674 | 2.285 |
| Coprococcus | 0.97 | -0.0326 | 0.42 |
| Adlercreutzia | 0.97 | 0.0331 | 0.68 |

**(B)**

**(A)**


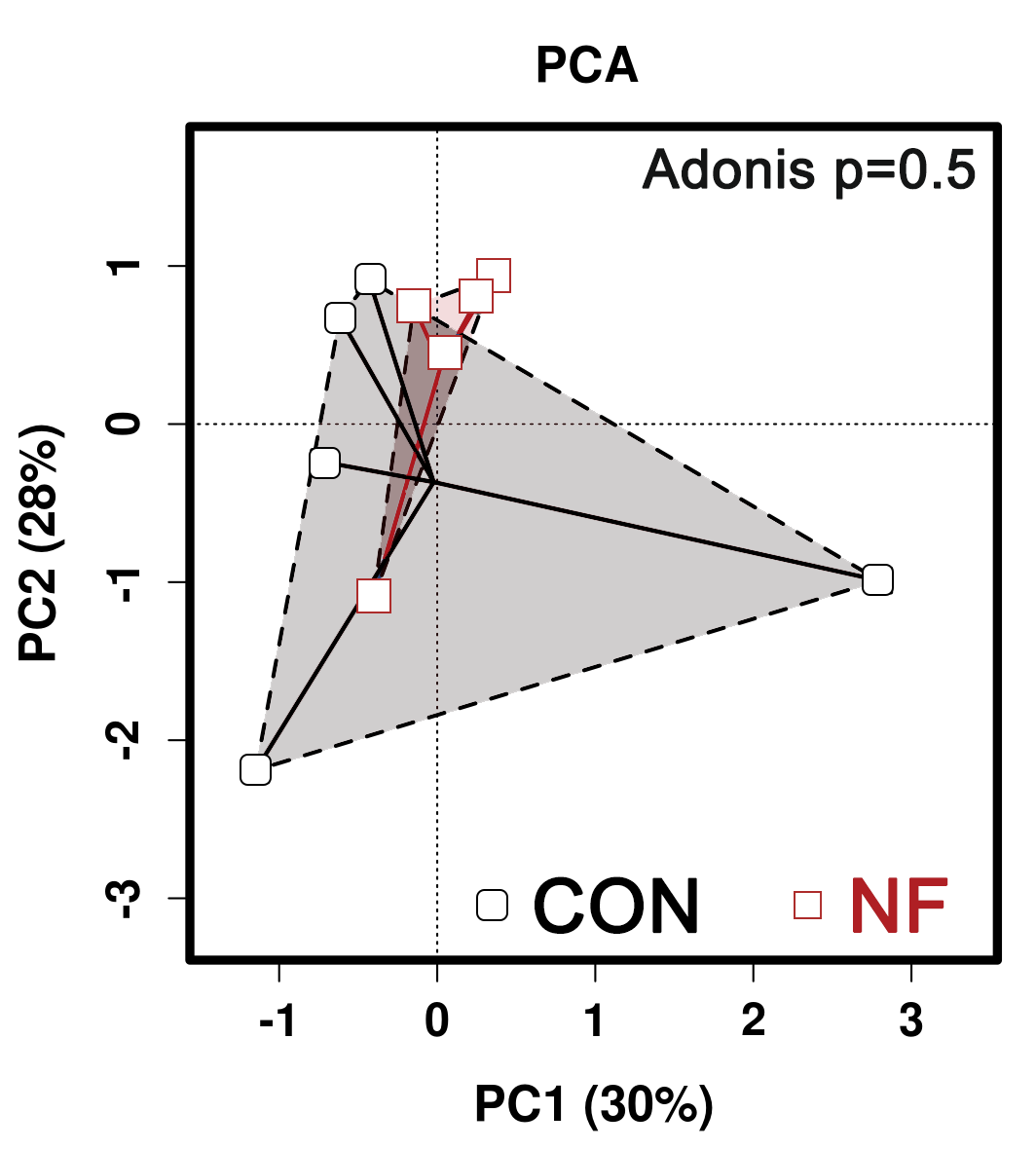

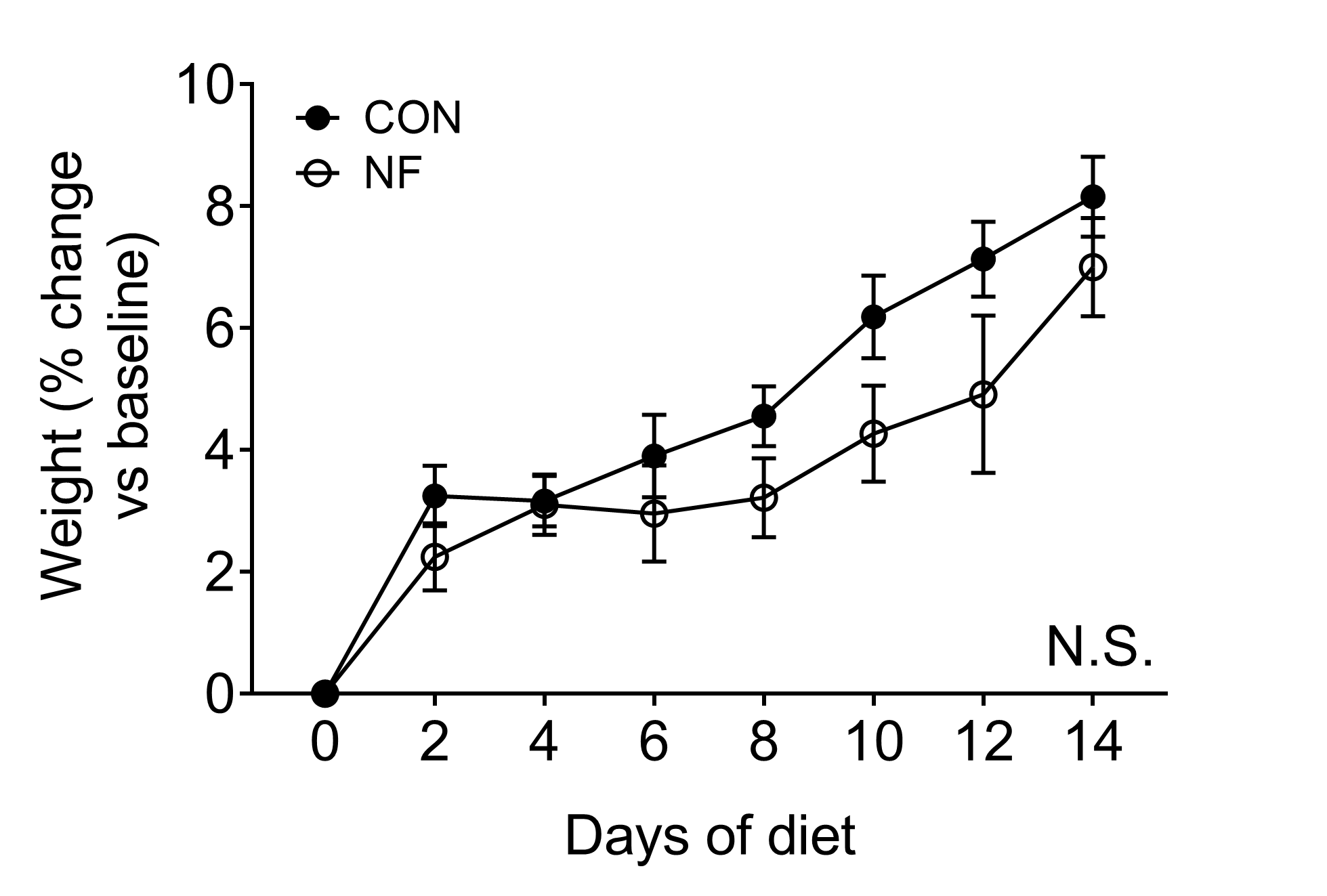


**Supplemental Figure 1: No difference in microbiota composition of mice prior to experimental diets or weight change after 2 weeks of experimental diet feeding.** (**A**) Comparable microbiota composition between the mice randomly assigned to experimental groups at the start of experiment. Dots represent individual mice, N ≥ 4. (**B**) Comparable weight gain over the 2 weeks of experimental diet between the mice randomly assigned to control or no-fibre feeding. Data displayed as mean ± SEM. N ≥ 50 individual mice per group. Two-way ANOVA was performed for all data across all time point and N.S. denotes no statistical significance.


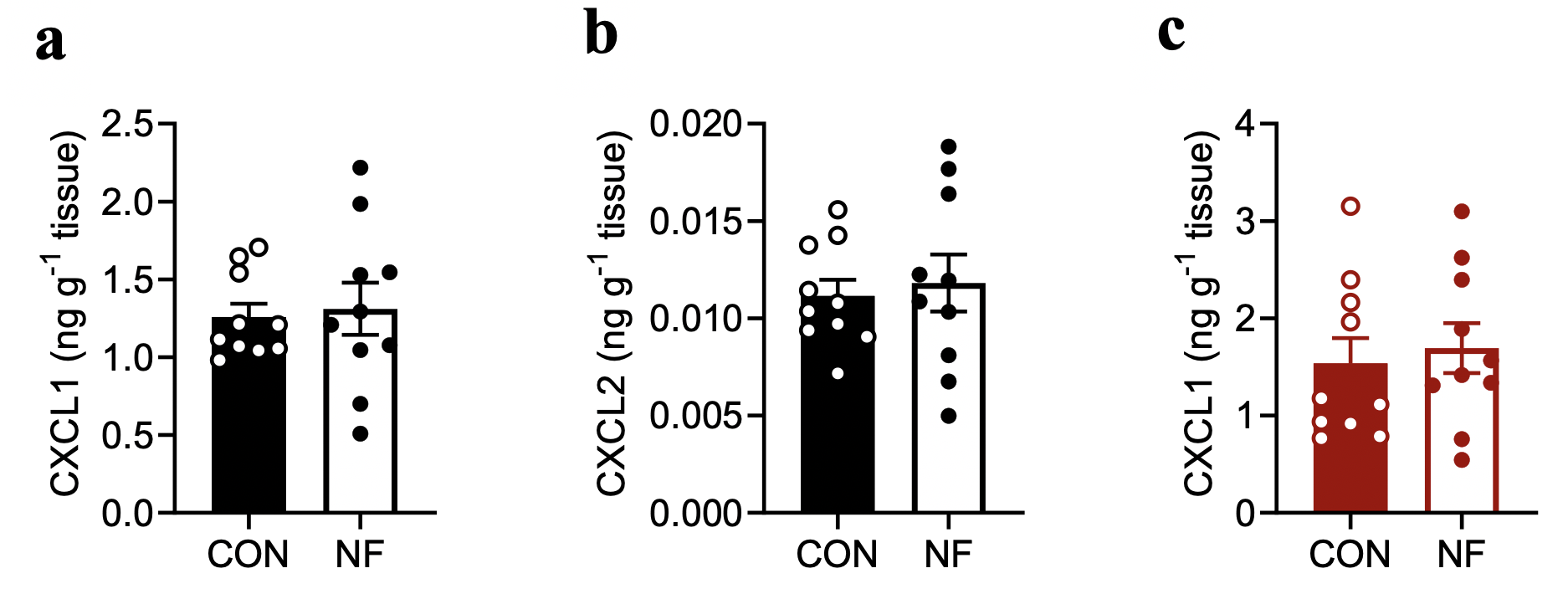


**(A)**

**(C)**

**(B)**

**Supplemental Figure 2: Protein levels of CXCL1 and CXCL2 in the colon.** Colonic protein levels of CXCL1 (**A**) and CXCL2 (**B**) following 2 weeks of the experimental diet were determined via ELISA. In addition, comparable colonic protein level of CXCL1 (**C**) was found in mice following the 2-week experimental diet feeding and DSS-induced colitis. Data displayed as mean ± SEM. One independent experiment was performed, N ≥ 9 individual mice per group.

**(A)**

**(B)**







**Supplemental Figure 3: Correlation of gut microbiota with DAI.** Negative correlation of the abundance and presence of Verrucomicrobia at the phylum level (**A**) and Akkermansia at the genus level (**B**) with disease activity index (DAI) following DSS-induced colitis was found in the no-fibre feeding cohort. One independent experiment was performed, N = 4 individual mice per group.


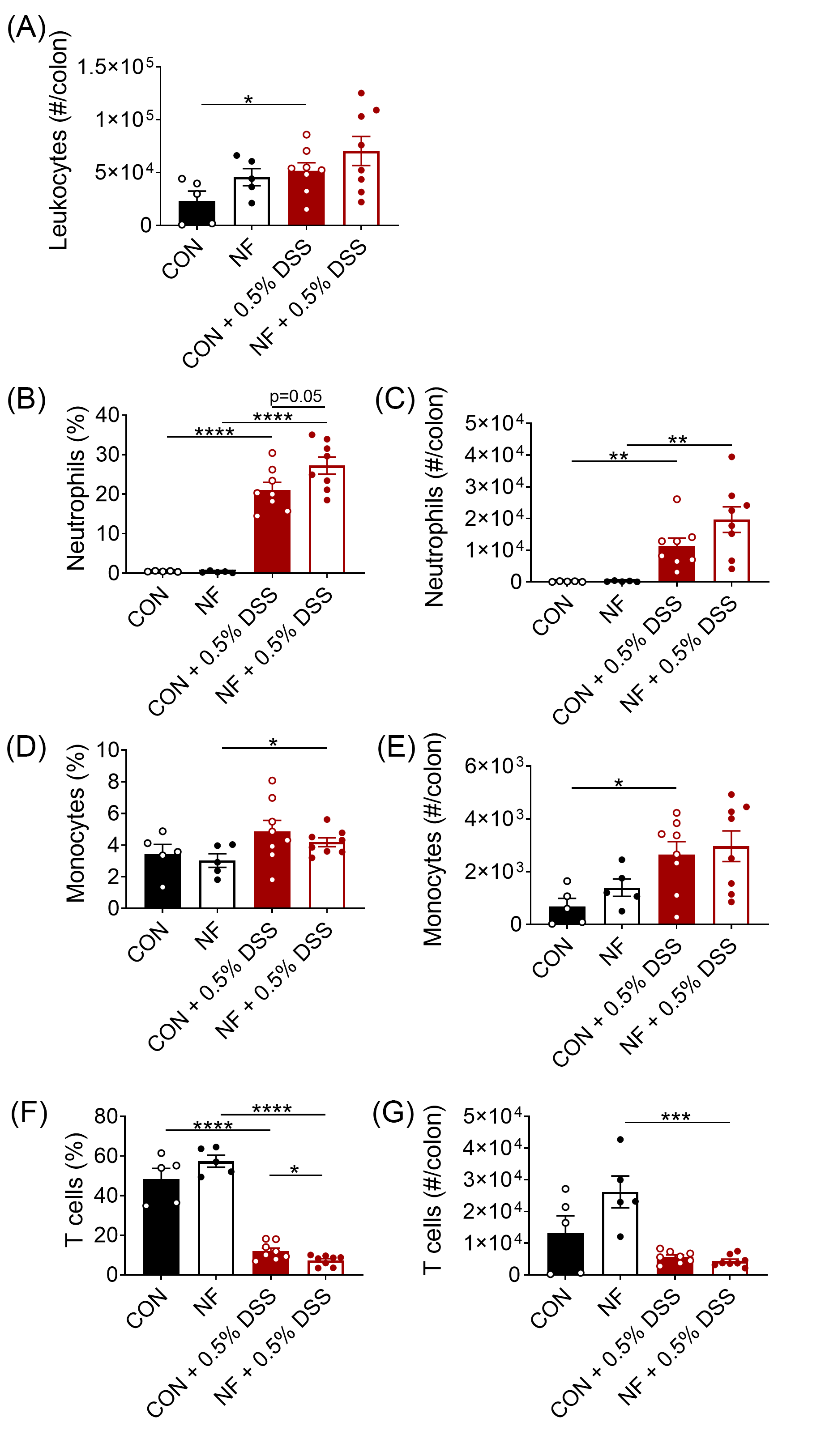


**Supplemental Figure 4: Colonic leukocyte populations in mice fed NF diet following DSS-induced colitis.** WT mice were fed either a control (CON) or no fibre (NF) diet for 14 days prior to and during 7 days of 0.5% DSS-induced colitis. At experimental endpoint, the colon was collected, and leukocytes quantitated by flow cytometry using fluorescent counting beads. (**A**) Total numbers of colonic leukocytes, the proportions and numbers of (**B, C**) neutrophils, (**D, E**) monocytes, and (**F, G**) T cells were enumerated. N = 5-8 per group. Dots represent individual mouse. Lines represent mean ± SEM. Student’s *t* test was performed, comparing CON vs NF; CON vs CON + 0.5% DSS; NF vs NF + 0.5% DSS; CON + 0.5% DSS vs NF + 0.5% DSS. **P* < 0.05, ***P* < 0.01, ****P* < 0.001, *****P* < 0.0001.


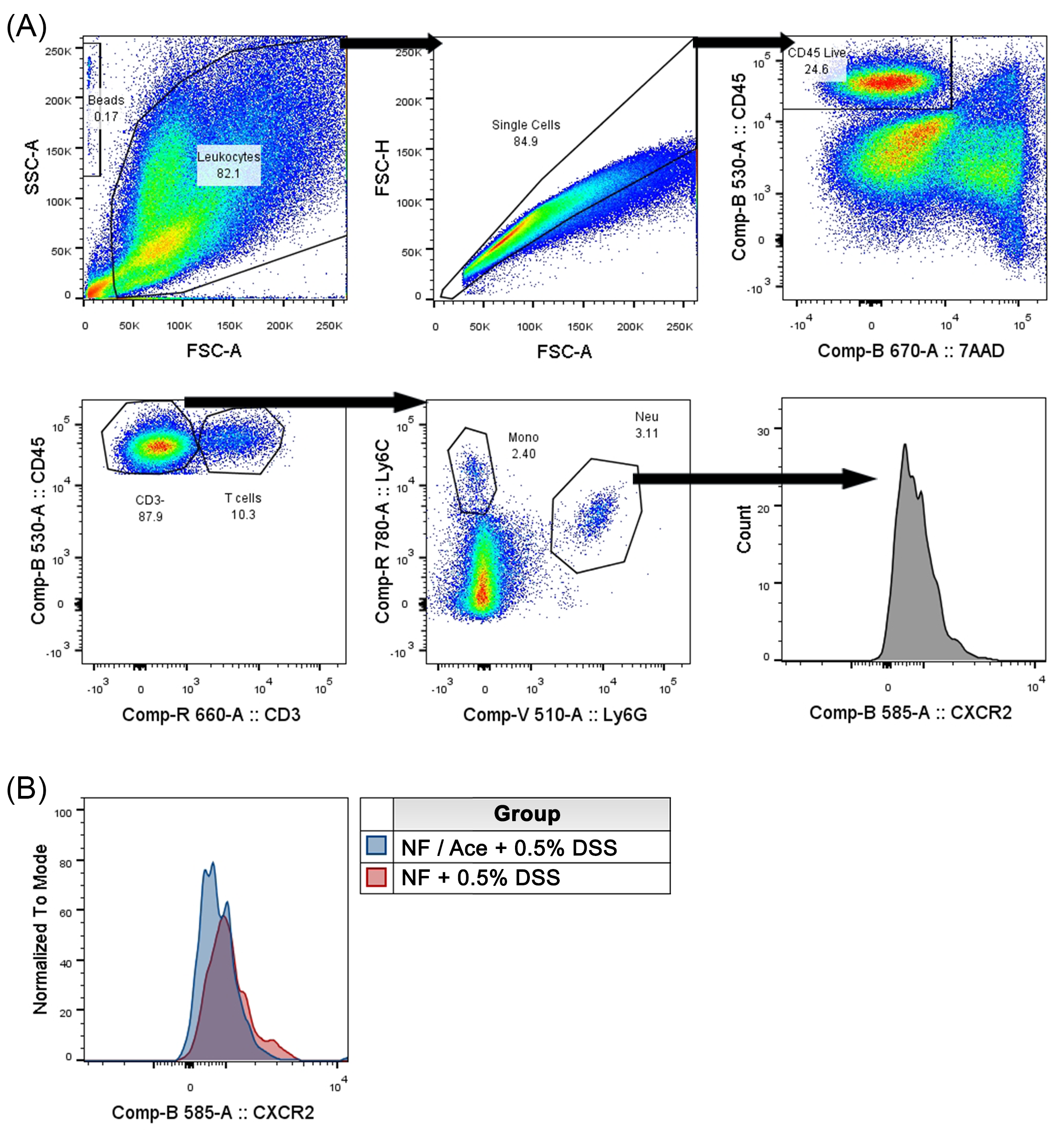


**Supplemental Figure 5: Representative flow cytometry plots.** (**A**) Gating strategy employed to identify colonic neutrophils (CD45^+^CD3^-^Ly6G^+^) for the study as well as for the investigation of cell surface expression of CXCR2. (**B**) Representative histogram demonstrating reduced CXCR2 surface expression on colonic neutrophils in no-fibre fed mice post-DSS that were supplemented with acetate compared to those that were not.
